# Non-invasive prediction of invasive lung adenocarcinoma and high-risk histopathological characteristics in resectable early-stage adenocarcinoma by [18F]FDG PET/CT radiomics-based machine learning models: a prospective cohort study

**DOI:** 10.1097/JS9.0000000000003464

**Published:** 2025-09-10

**Authors:** Xinghui Cao, Zhilei Lv, Yan Li, Minglei Li, Yuanyang Hu, Mengyuan Liang, Jingjing Deng, Xueyun Tan, Sufei Wang, Wei Geng, Juanjuan Xu, Ping Luo, Mei Zhou, Wenjing Xiao, Mengfei Guo, Jiatong Liu, Qi Huang, Shengqing Hu, Yice Sun, Xiaoli Lan, Yang Jin

**Affiliations:** a Department of Respiratory and Critical Care Medicine, Hubei Province Clinical Research Center for Major Respiratory Diseases, Key Laboratory of Pulmonary Diseases of National Health Commission, Union Hospital, Tongji Medical College, Huazhong University of Science and Technology, Wuhan, Hubei, China; b Hubei Province Engineering Research Center for Tumor-Targeted Biochemotherapy, MOE Key Laboratory of Biological Targeted Therapy, Union Hospital, Tongji Medical College, Huazhong University of Science and Technology, Wuhan, Hubei, China; c Department of Pathology, Union Hospital, Tongji Medical College, Huazhong University of Science and Technology, Wuhan, Hubei, China; d Hubei Province Key Laboratory of Biological Targeted Therapy, Union Hospital, Tongji Medical College, Huazhong University of Science and Technology, Wuhan, Hubei, China; e Department of Translational Medicine Center, Union Hospital, Tongji Medical College, Huazhong University of Science and Technology, Wuhan, Hubei, China; f Department of Nuclear Medicine, Union Hospital, Tongji Medical College, Huazhong University of Science and Technology, Wuhan, Hubei, China; g Department of Respiratory and Critical Care Medicine, Renmin Hospital of Wuhan University, Wuhan, Hubei, China; h Hubei Key Laboratory of Molecular Imaging, Wuhan, China

**Keywords:** cohort study, lung adenocarcinoma, multicenter, PET/CT, radiomics, thoracic surgery

## Abstract

**Background:**

Precise preoperative discrimination of invasive lung adenocarcinoma (IA) from preinvasive lesions (adenocarcinoma *in situ* [AIS]/minimally invasive adenocarcinoma [MIA]) and prediction of high-risk histopathological features are critical for optimizing resection strategies in early-stage lung adenocarcinoma (LUAD).

**Methods:**

In this multicenter study, 813 LUAD patients (tumors ≤3 cm) formed the training cohort. A total of 1709 radiomic features were extracted from the PET/CT images. Feature selection was performed using the max-relevance and min-redundancy algorithm and least absolute shrinkage and selection operator. Hybrid machine learning models integrating [18F]FDG PET/CT radiomics and clinical–radiological features were developed using H2O.ai AutoML. Models were validated in a prospective internal cohort (*N* = 256, 2021–2022) and external multicenter cohort (*N* = 418). Performance was assessed via area under the curve (AUC), calibration, decision curve analysis (DCA), and survival assessment.

**Results:**

The hybrid model achieved AUCs of 0.93 (95% CI: 0.90–0.96) for distinguishing IA from AIS/MIA (internal test) and 0.92 (0.90–0.95) in external testing. For predicting high-risk histopathological features (grade-III, lymphatic/pleural/vascular/nerve invasion, and spread through air spaces), AUCs were 0.82 (0.77–0.88) and 0.85 (0.81–0.89) in internal/external sets. DCA confirmed superior net benefit over CT model. The model stratified progression-free (*P* = 0.002) and overall survival (*P* = 0.017) in the TCIA cohort.

**Conclusion:**

PET/CT radiomics-based models enable accurate non-invasive prediction of invasiveness and high-risk pathology in early-stage LUAD, guiding optimal surgical resection.

**HIGHLIGHTS**

- Innovative PET/CT-based models non-invasively predict invasive lung cancer and high-risk features.
- Clinically validated in large multicenter cohorts to guide precise surgical decisions for patients with early-stage disease.
- Prospective validation confirms robust performance in predicting tumor aggressiveness and survival outcomes.
- Machine learning models identify biological pathways linked to LUAD progression.

## Introduction

The International Association for the Study of Lung Cancer, American Thoracic Society, and European Respiratory Society (IASLC/ATS/ERS) have proposed a multidisciplinary classification of lung adenocarcinoma (LUAD)^[1]^, encompassing adenocarcinoma-*in-situ* (AIS), minimally invasive adenocarcinoma (MIA), and invasive adenocarcinoma (IA). AIS and MIA are curable through sublobar resection, and patients afflicted with either of these conditions exhibit a 10-year disease-free survival rate of nearly 100%. The JCOG0802 clinical trial^[2]^ demonstrated that segmentectomy is as efficacious as lobectomy and correlates with enhanced lung function recovery in patients with small-peripheral (tumors ≤2 cm) non-small-cell lung cancer (NSCLC), irrespective of subtypes. Nevertheless, high-risk histopathological attributes were discovered to be independently associated with the risk of recurrence in patients undergoing surgical resection, particularly segmental resection and tumor dimensions exceeding 2 cm^[3-7]^. Consequently, it is crucial that these high-risk attributes are accurately identified non-invasively prior to surgery, and that the optimal surgical approach and lymph node dissection area are determined, particularly for patients with small-peripheral pulmonary nodules. This is essential for securing a favorable prognosis.

Several conventional methodologies have been employed to differentiate invasive LUAD and predict invasiveness and metastasis potential of LUAD, including CT imaging signs and intraoperative frozen sections (FS). Physicians utilize CT scans to ascertain the pathological classification and high-risk histopathological attributes of pulmonary nodules, including tumor dimensions, consolidation rate of primary tumors, and CT-signs, such as spiculation signs, all of which are highly subjective with undefined accuracy. Preoperative cytological specimens or small biopsies of primary tumor play a limited role in determining invasiveness or metastasis potential due to limited sampling or heterogeneity of the tumor. The precise diagnosis of FS is an effective method for directing the resection of peripheral small-sized LUAD^[8]^. However, this could be challenging for general pathologists practicing in remote or suburban hospitals, where resource limitations, high workloads, and reduced access to subspecialty expertise may contribute to higher diagnostic variability in FS interpretation^[9,10]^. Therefore, a novel and more readily available strategy should be developed to guide surgical resection.

Recently, several studies suggest that metabolic parameters acquired using positron emission tomography with 2-deoxy-2-[fluorine-18] fluoro-d-glucose integrated with CT ([18F]FDG PET/CT) are effective in predicting pathological classification, invasion and metastasis potential^[11–13]^. Furthermore, PET/CT radiomics could rapidly capture quantitative and textural imaging features related to morphology and metabolism. Therefore, we propose that PET/CT radiomics may be advantageous for precisely predicting the invasiveness and metastasis potential of early-stage LUAD. Artificial intelligence could further objectively evaluate clinical–radiological data, resulting in more accurate diagnoses of diseases and reduced effort required in routine clinical practice. Therefore, we believe that machine learning models based on PET/CT radiomics could be a suitable and effective guide for surgical strategies in lung cancer.

Furthermore, imaging characteristics provide a plethora of information regarding cancer genotypes^[14]^. However, the correlation between radiomics features and genomic alterations remains largely unknown, particularly in the context of PET/CT. Reliable machine learning models were established based on PET/CT radiomic features and various clinical indicators, with the aim of accurately predicting the pathological classification and high-risk histopathological characteristics of early-stage LUAD prior to surgery. The latest clinical study, JCOG0802^[2,15]^, reported that segmentectomy is equivalent to lobectomy in efficacy and correlates with improved lung function recovery in individuals with small-peripheral (lung nodules ≤2 cm) NSCLC. The assessment of the predictive efficacy of our models concentrated on the subgroup exhibiting tumor size ≤2 cm and ground-glass nodules (GGN), given the current clinical controversy regarding the selection of surgical modality for small-peripheral lung nodules^[2,16]^. Moreover, we integrated radiomics with RNA sequencing data pertaining to high-risk histopathological characteristics with the objective of elucidating the underlying biological mechanisms that underpin the pathological evolution. This study has been reported in line with the STROCSS criteria^[17]^.

## Methods

### Participant inclusion

**Figure 1.**
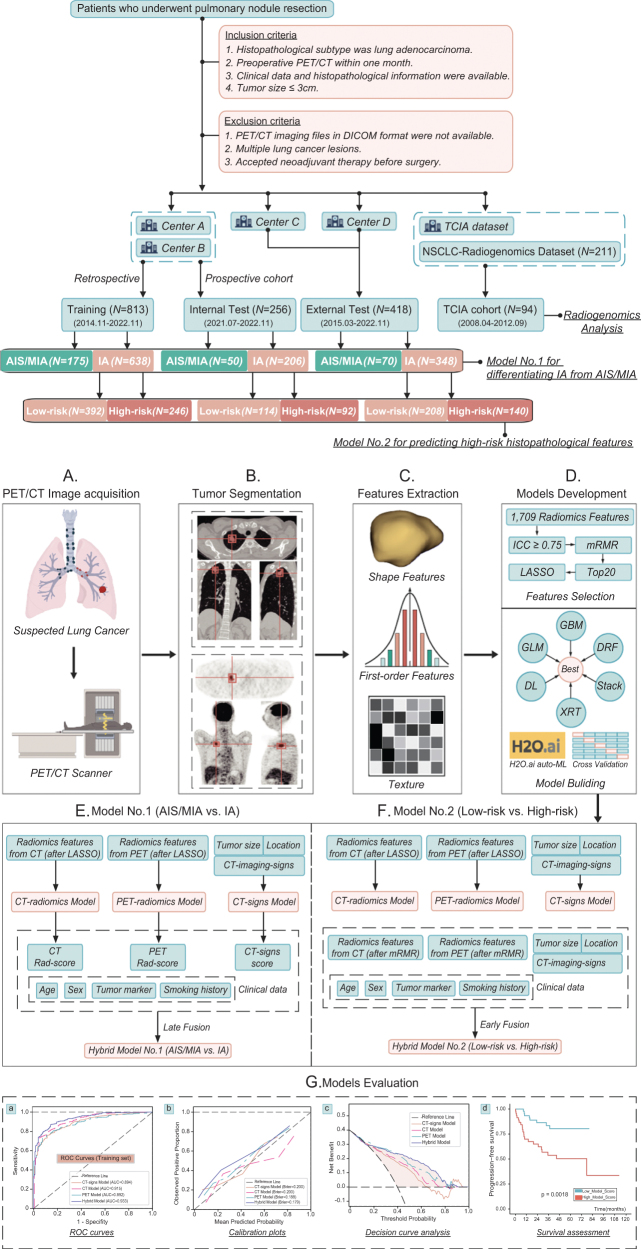
Study flowchart (patient selection and model development). (A) PET/CT Images acquisition. (B) Tumor segmentation. (C) Radiomics features extraction. (D)–(F) Models Development. (G) Models evaluation. AIS, adenocarcinoma *in situ*; MIA, minimally invasive adenocarcinoma; IA, invasive adenocarcinoma; DL, deep learning; GBM, gradient boosting machine; GLM, generalized linear model; DRF, distributed random forest; XRT, extremely randomized trees; Stack, stacking ensemble model. Model No. 1 and Model No. 2 were constructed to serve two distinct objectives: (1) to differentiate IA from AIS/MIA and (2) to predict high-risk histopathological features in early-stage LUAD, which include grade-III subtype, lymphatic metastasis, pleural invasion, vascular invasion, nerve invasion, and spread through air spaces (STAS). Early fusion and late fusion were utilized to develop these two models.

In total, 813 LUAD patients with tumors ≤3 cm who underwent curative-intent resection between November 2014 and November 2022 from Center-A and Center-B were included in the training set. Model validation was performed using a prospective cohort (*N* = 256) from July 2021 to November 2022, serving as the internal test set. An additional external multicenter cohort (*N* = 418) from Center-C and Center-D was also utilized (Fig. 1). Inclusion criteria were: (1) histopathological subtypes were LUAD, (2) underwent preoperative [18F]FDG PET/CT scan within 1 month, (3) clinical data and histopathological information were available, and (4) tumor size ≤3 cm. Exclusion criteria included: (1) unavailability of PET/CT imaging files, (2) multiple lung cancer lesions, and (3) accepted neoadjuvant therapy before surgery. The NSCLC-Radiogenomics collections (*N* = 94) from the Cancer Imaging Archive (TCIA) was incorporated to further assess the performance of hybrid model^[18]^. This study and protocol were registered at ClinicalTrials.gov. Ethical approval was obtained from Wuhan Union Hospital Ethics Committee.

### Clinical–radiological characteristics evaluation

This research compiled a comprehensive array of clinical and radiological characteristics, inclusive age, gender, smoking history, tumor markers, and genetic mutations. Two seasoned experienced radiologists meticulously assessed the CT imaging signs with the primary lung cancer. This evaluation encompassed the presence of solid or pure/mixed GGN; signs such as spiculation, lobulation, pleural changes, vacuole sign, air bronchogram, vascular convergence, and calcification were also assessed. The PET semi-quantitative metabolic parameters (SUVmax, SUVmean, and TLG) associated with [^18^F]FDG uptake for tumors were calculated using the “Quantitative Indices Tool” module of 3D-Slicer software (version 5.2.2)^[19]^.

### Pathological classification

Surgically excised tissues were routinely processed and evaluated in the department of pathology. The primary pathological classification divided adenocarcinoma into AIS, MIA, and IA.

Invasive non-mucinous adenocarcinoma were further classified into three grades based on the predominant and high-grade patterns: grade-I (lepidic predominant adenocarcinoma with no, or less than 20% of high-grade patterns), grade-II (acinar or papillary predominant adenocarcinoma with no, or less than 20% of high-grade patterns), and grade-III (any tumor with 20% or more of high-grade patterns, such as solid, micropapillary, or complex gland). Histopathological statuses including lymphatic metastasis, pleural invasion, vascular invasion, nerve invasion, and spread through air spaces were also evaluated based on final pathology (FP). Then, patients with either grade-III or any above status were designated as high-risk histopathological characteristics group.

### Intra-operation FS evaluation

Following tumor was being extirpated by surgical resection, pathologists promptly diagnosed FS of the specimens. The specimen was sliced at the largest diameter of the tumor for sampling. Typically, two sections were procured from each specimen for intraoperative diagnosis. If the lesions were diagnosed as adenocarcinoma, FS were further stratified into AIS, MIA, and IA.

### Tumor segmentation, radiomic features extraction, and screening

A radiologist delineated region of interest (ROI) on CT and PET imaging by utilizing 3D-Slicer software. A total of 1,709 radiomics features were extracted from both CT and PET images. The Image acquisition protocol and detailed extraction methods are provided in *Supplementary materials.* Three steps were employed for radiomics feature screening. Initially, a radiologist performed repeated the segmentation of 50 randomly selected patients after 2 weeks to evaluate the intra-observer reliability of extracted radiomics features. Moreover, another radiologist independently delineated the ROI for assessing inter-observer reliability. Radiomics features with intraclass correlation coefficient (ICC) less than were eliminated. Subsequently, the max-relevance and min-redundancy (mRMR) algorithm were utilized for filtering the top 20 features. Lastly, radiomics features were refined with Least absolute shrinkage and selection operator (LASSO) logistic regression via five-fold cross validation.

### Machine learning models development and validation

Our objective was to develop models for the subsequent two tasks: distinguishing (a) AIS/MIA from IA, (b) high-risk pathological characteristics group from low-risk group. We utilized the synthetic minority over-sampling technique^[20]^ to address imbalance issue of the training set. CT-signs, CT, PET radiomics, and hybrid models were developed by H_2_O Automatic Machine Learning (auto-ML)^[21]^, which enables for automatic training and tuning of numerous models within a designated timeframe. Various cutting edge supervised and unsupervised algorithms including deep learning, gradient boosting machine, generalized linear model, distributed random forest, extremely randomized trees, and stacking ensemble models were implemented in training set for model development. The steps for developing CT-signs, CT, PET radiomics, and hybrid models were detailed in *Supplementary materials*. The model with the optimal predictive performance among those automatically trained by H_2_O auto-ML was selected based on the area under the curve (AUC) and logloss for test set. The predictive performance was evaluated using AUC, sensitivity, specificity, accuracy, and Brier score. Additionally, decision curve analysis (DCA) and calibration plots were conducted to assess the clinical impact of the model. For subgroup analysis, the evaluation of the predictive effect of our model focused on the subgroup with tumor ≤2 cm and GGN subgroup, due to the current clinical debate on the choice of surgical modality for small-peripheral lung nodules^[2,16]^.

### Survival assessment

Clinical data encompassed, where available, PET/CT date, date of recurrence, date of last known alive, survival status, and recurrence status were obtained from the TCIA set^[17]^. Those with unconfirmed deaths were censored at the last recorded date of the medical procedure. Kaplan–Meier curves with the log-rank test were used to assess the progression-free survival (PFS) and overall survival (OS).

### Bioinformatics analysis

RNA-sequencing data of NSCLC-Radiogenomics collection (GSE103584) from samples of surgically excised tumor tissue was utilized for radiogenomics analysis to elucidate the underlying biological mechanisms of pathological evolution. Differentially expressed genes were identified using the edgR package (version 4.0.16, |log_2_FC| > 1.5 and FDR<0.05). Gene set variation analysis (GSVA) was conducted to determine relationships between genomics characterization and radiomics score by hallmark gene sets (https://www.gsea-msigdb.org). Single-sample gene set enrichment analysis (ssGSEA) was employed to quantify the prevalence of immune cell subtypes based on immune cell-related signatures^[22]^. Weighted gene co-expression network analysis (WGCNA) was conducted using the cutreeDynamic function of WGCNA package (version 1.72-5). The “salmon module” (automatically named by the WGCNA algorithm) was highlighted for its strong correlation with PET metabolic parameters (SUVmax/TLG) and was further analyzed for biological pathway enrichment. The Sankey bubble map was plotted using SRplot, an online platform^[23]^.

### Statistical analyses

The normal distribution was ascertained utilizing the Shapiro–Wilk test, and homogeneity of variance between cohorts was screened using Levene’s test. Student’s *t*-test was used for the comparison of normally distributed data. Inter-group comparisons for baseline characteristics were assessed using Kruskal–Wallis for continuous variables and Chi-square/Fisher’s exact tests for categorical variables, with significance threshold *P* < 0.05. Statistical analyses were conducted using R (version 4.3.1) and Python (version 3.8.16).

## Results

### Patient characteristics

**Figure 2.**
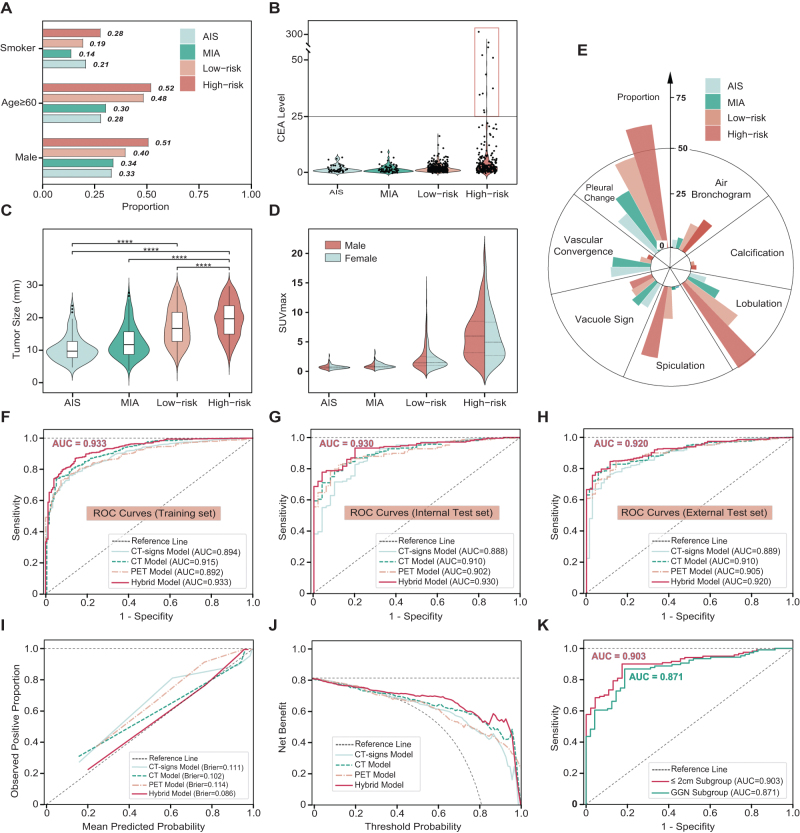
**Performance of prediction models for differentiating AIS/MIA from IA.** We performed comparative analyses of the following among the pathological subtypes of lung adenocarcinoma: **(A)** demographics (sex, age, and smoking status), **(B)** CEA levels, **(C)** CT-measured tumor size, and **(D**) SUVmax. Statistically significant differences between groups are indicated by asterisks (****) in the relevant figures. **(E)** Stacked bar chart showing the proportion of various CT imaging signs across different pathological classifications. ROC curves of the CT-signs, CT radiomics, PET radiomics, and hybrid models for differentiating AIS/MIA from IA in **(F)** Training set, **(G) i**nternal test set, and **(H)** external test set. **(I).** Calibration plots of the CT, PET, and hybrid models show the consistency between the mean predicted probability of IA and actual proportion in the internal test set. (**J)** Decision curve analysis of models for internal test set. **(K).** ROC curves of models for GGN or Tumors ≤2 cm subgroups in internal set.

The study flowchart was shown in Fig. 1. A total of 1581 patients were included in this study, which was composed of the training set (*N* = 813) and prospectively internal test set (*N* = 256) and external test set (*N* = 418). The patients from the open repository served as TCIA set (*N* = 94). The baseline characteristics of all patients were detailed in Table 1. The proportion of pathological subtypes (AIS, MIA, Low-risk IA, and High-risk IA) in training and test datasets did not exhibit a significant divergence (*P* = 0.317). IA (especially high-risk group) exhibited a higher proportion of elderly males and smoker (Fig. 2A), higher CEA level (Fig. 2B), larger tumor diameters (Fig. 2C), and elevated SUVmax (Fig. 2D) when compared to AIS and MIA groups. The CT-signs associated with IA subtypes demonstrated a higher prevalence of spiculation, lobulation, pleural changes, and air bronchograms (Fig. 2E). It is worth mentioning that AIS and MIA groups showed no calcification on CT images. An overview of the clinical, radiological, and pathological profiles according to the multidisciplinary classification of LUAD is illustrated in Supplemental Digital Content Table S1, available at, http://links.lww.com/JS9/F100.

**Table 1:** Baseline patients characteristics

	Training set	Internal test set	External test set	TCIA set	
Characteristics	(*N* = 813)	(*N* = 256)	(*N* = 418)	(*N* = 94)	*P*-value*
Sex (female)	478 (58.8)	146 (57.0)	239 (57.2)	40 (42.6)	0.809
Age (years)	57.0 [52.0, 64.0]	60.0 [53.0, 67.0]	59.0 [53.0, 65.8]	67.0 [62.0, 72.8]	<0.001
Lifetime smoker	155 (19.1)	62 (24.2)	97 (23.2)	69 (73.4)	0.099
CEA level	2.1 [1.3, 3.5]	2.1 [1.3, 3.4]	2.2 [1.4, 3.3]	–	0.812
CA19-9 level	6.2 [3.1, 13.1]	5.6 [2.9, 11.0]	4.6 [2.6, 11.3]	–	0.075
CA125 level	10.5 [8.3, 15.6]	9.85 [7.5, 14.3]	11.0 [8.4, 15.8]		0.312
CA15-3 level	7.9 [6.4, 11.7]	10.4 [6.8, 17.3]	8.7 [6.2, 14.0]	–	0.111
CA74-2 level	2.0 [1.1, 3.8]	1.76 [1.23, 3.51]	1.6 [1.2, 2.5]	–	0.067
Tumor size, cm	1.7 [1.2, 2.1]	1.7 [1.3, 2.2]	1.7 [1.3, 2.3]	1.9 [1.4, 2.3]	0.089
Location (%)					0.733
LLL	109 (13.4)	37 (14.5)	56 (13.4)	13 (13.8)	
LUL	218 (26.8)	77 (30.1)	102 (24.4)	25 (26.6)	
RLL	129 (15.9)	43 (16.8)	64 (15.3)	7 (7.4)	
RML	57 (7.0)	18 (7.0)	35 (8.4)	11 (11.7)	
RUL	300 (36.9)	81 (31.6)	161 (38.5)	38 (40.4)	
Nodule type					0.915
GGN	481 (59.2)	154 (60.2)	252 (60.3)	44 (46.8)	
Solid/vesicle	332 (40.8)	102 (39.8)	166 (39.7)	50 (53.2)	
SUVmax	1.6 [0.9, 4.3]	1.6 [1.0, 4.0]	1.9 [1.0, 4.4]	3.0 [1.5, 5.8]	0.277
SUVmean	1.0 [0.6, 2.0]	0.9 [0.6, 2.0]	1.0 [0.6, 2.0]	1.5 [1.1, 2.3]	0.660
TLG	2.9 [0.7, 9.6]	3.5 [1.0, 10.9]	4.3 [1.1, 12.2]	6.6 [3.7, 19.7]	0.041
EGFR mutation					<0.001
Mutant	147 (18.1)	76 (29.7)	52 (12.4)	21 (22.3)	
Wildtype	53 (6.5)	34 (13.3)	17 (4.1)	61 (64.9)	
Unknown	613 (75.4)	146 (57.0)	349 (83.5)	12 (12.8)	
KRAS mutation					<0.001
Mutant	5 (0.6)	9 (3.5)	1 (0.2)	20 (21.3)	
Wildtype	195 (24.0)	101 (39.5)	68 (16.3)	59 (62.8)	
Unknown	613 (75.4)	146 (57.0)	349 (83.5)	15 (16.0)	
ALK translocation					<0.001
Mutant	2 (0.2)	5 (2.0)	1 (0.2)	–	
Wildtype	198 (24.4)	105 (41.0)	68 (16.3)	75 (79.8)	
Unknown	613 (75.4)	146 (57.0)	349 (83.5)	19 (20.2)	
Pathology subtype					0.317
AIS	59 (7.3)	14 (5.5)	24 (5.7)	–	
MIA	116 (14.3)	36 (14.1)	46 (11.0)	–	
Low-risk IA	392 (48.2)	114 (44.5)	208 (49.8)	–	
High-risk IA^a^	246 (30.3)	92 (35.9)	140 (33.5)	–	
Lymphatic metastasis					0.821
N0	716 (88.1)	226 (88.3)	361 (86.4)	60 (63.8)	
N1	16 (2.0)	4 (1.6)	6 (1.4)	3 (3.2)	
N2	64 (7.9)	20 (7.8)	38 (9.1)	4 (4.3)	
Unknow	17 (2.1)	6 (2.3)	13 (3.1)	27 (28.7)	
Surgical procedure					0.258
Lobectomy	576 (70.8)	176 (68.8)	308 (73.7)	–	
Segmentectomy	180 (22.1)	68 (26.6)	88 (21.1)	–	
Wedge	57 (7.0)	12 (4.7)	22 (5.3)	–	

Data are presented as *n* (%) or median [interquartile range]. LLL, left lower lobe; LUL, left upper lobe; RLL, right lower lobe; RML, right middle lobe; RUL, right upper lobe; GGN, ground-glass nodules; SUV, standardized uptake value; TLG, total lesion glycolysis.

^a^ Patients characterized by grade-III IA or any histopathological features, including lymphatic metastasis, pleural invasion, vascular invasion, nerve invasion, and spread through air spaces, are categorized as high-risk histopathological characteristics.

^*^ The “*P*-value” column indicates the inter-group differences observed among the training set, internal test set, and external test set.

### Performance of prediction models for distinguishing AIS/MIA from IA

As it is universally acknowledged that LAUD with tumor size >3 cm were invariably IA, we excluded patients with tumor size >3 cm for developing models for distinguishing AIS/MIA from IA. Subsequently, we developed a CT-signs model based on demographics (sex, age, and smoker) and CT-imaging-signs (GGN, spiculation, lobulation, pleural changes, vacuole sign, air bronchogram, vascular convergence, and calcification) to differentiate AIS/MIA from IA. For the CT-signs model, the AUC values were 0.894, 0.888, and 0.889 for the training, internal test, and external test datasets, respectively (Fig. 2F–H). The radiomics features were selected by the ICC, mRMR algorithm, and LASSO regression. Then selected radiomics and clinical–radiological characteristics were amalgamated for machine learning model training. The performance of hybrid model on the internal set was evaluated by AUC, sensitivity, specificity, accuracy, and Brier score in predicting IA. Values obtained were favorable at 0.93 (0.90–0.96), 0.87 (0.84–0.90), 0.86 (0.80–0.91), 0.87 (0.84–0.89), and 0.09 surpassing CT-signs, CT, and PET radiomics model (Fig. 2G, Table 2). The AUC of hybrid model was documented as 0.920 for external test set (Fig. 2H). The calibration curve of hybrid model showed good agreement between prediction probabilities and actual observation probabilities of IA for internal test (Fig. 2I). The net benefit of hybrid model was superior to those three models for most thresholds according to DCA (Fig. 2J).

**Table 2:** The predictive performance of CT-signs models and hybrid models

	*Models No. 1 (AIS/MIA vs IA)*		*Models No. 2 (low-risk vs. high-risk)*	
Models	CT-signs Model	Hybrid Model	CT-signs Model	Hybrid Model
Training set				
AUC	0.89 (0.87–0.92)	0.93 (0.91–0.95)	0.83 (0.79–0.86)	0.87 (0.84–0.90)
Sensitivity	0.76 (0.73–0.79)	0.87 (0.84–0.90)	0.84 (0.80–0.88)	0.83 (0.80–0.86)
Specificity	0.89 (0.84–0.93)	0.86 (0.80–0.91)	0.71 (0.66–0.76)	0.80 (0.77–0.83)
Accuracy	0.79 (0.76–0.81)	0.87 (0.84–0.89)	0.76 (0.73–0.80)	0.81 (0.78–0.84)
Brier score	0.12	0.09	0.16	0.14
Internal test set				
AUC	0.89 (0.84–0.94)	0.93 (0.90–0.96)	0.73 (0.66–0.80)	0.82 (0.77–0.88)
Sensitivity	0.83 (0.77–0.88)	0.78 (0.72–0.83)	0.84 (0.75–0.91)	0.76 (0.70–0.82)
Specificity	0.80 (0.69–0.92)	0.96 (0.90–1.00)	0.57 (0.48–0.66)	0.78 (0.72–0.84)
Accuracy	0.82 (0.77–0.87)	0.81 (0.76–0.86)	0.69 (0.63–0.75)	0.77 (0.72–0.83)
Brier score	0.11	0.09	0.21	0.18
Internal test set (Tumor size ≤2 cm subgroup)				
AUC	0.86 (0.80–0.92)	0.90 (0.85–0.94)	0.72 (0.63–0.82)	0.80 (0.73–0.87)
Sensitivity	0.88 (0.82–0.93)	0.90 (0.85–0.95)	0.77 (0.65–0.88)	0.65 (0.57–0.74)
Specificity	0.74 (0.60–0.85)	0.83 (0.71–0.93)	0.66 (0.54–0.78)	0.84 (0.77–0.90)
Accuracy	0.84 (0.78–0.89)	0.88 (0.83–0.93)	0.71 (0.62–0.79)	0.76 (0.68–0.84)
Brier score	0.15	0.11	0.22	0.19
External test set				
AUC	0.89 (0.85–0.92)	0.92 (0.90–0.95)	0.78 (0.73–0.83)	0.85 (0.81–0.89)
Sensitivity	0.76 (0.72–0.81)	0.78 (0.73–0.82)	0.71 (0.63–0.78)	0.79 (0.75–0.84)
Specificity	0.89 (0.80–0.96)	0.96 (0.91–1.00)	0.76 (0.71–0.82)	0.78 (0.74–0.83)
Accuracy	0.79 (0.74–0.82)	0.81 (0.77–0.85)	0.74 (0.70–0.79)	0.79 (0.74–0.83)
Brier score	0.12	0.10	0.18	0.15

Data within parentheses represent the 95% confidence intervals (CI). AUC refers to the area under the receiver operating characteristic (ROC) curve. The Brier score is a metric for assessing the calibration of the model. It quantifies the mean squared difference between predicted probabilities and observed outcomes; a lower Brier score signifies greater model accuracy

The FS was the definitive reference for the conclusive determination of the resection process. A total of 441 intraoperative FS for patients in clinical IA stage were executed (Supplemental Digital Content Figure S1, available at: http://links.lww.com/JS9/F100), and the overall concurrence rate of FS and FP was 90.0% (397/441) as per the IASLC/ATS/ERS classification. When the AIS and MIA groups were amalgamated into a single entity, the concurrence rate escalated to 93.0% (410/441). Significantly, multivariate regression analysis demonstrated that our hybrid model prediction outcome exhibited independent value from FS for differentiating IA from AIS/MIA (Table 3). The hybrid model score was the independent predictive factor for distinguishing between AIS/MIA and IA (*P* = 0.038). The importance score ranking of our model’s specific indicators for identifying IA vs AIS/MIA was shown in Supplemental Digital Content Figure S2A-D, available at: http://links.lww.com/JS9/F100 and Supplemental Digital Content Table S2, available at: http://links.lww.com/JS9/F100.

**Table 3:** Multivariable logistic regression analysis for distinguishing between AIS/MIA and IA

	Univariable		Multivariable	
Characteristics	OR	*P* value	OR	*P* value
Sex (male vs female)	1.07 (0.64–1.79)	0.804		
Age (≥60 vs <60 years)	2.33 (1.32–4.09)	0.003	1.33 (0.51–3.47)	0.554
Lifetime smoker	1.19 (0.65–2.17)	0.577		
CEA	1.34 (0.97–1.84)	0.073		
CA19-9	1.08 (0.99–1.18)	0.094		
CA125	1.00 (0.98–1.01)	0.807		
CA15-3	1.04 (0.91–1.13)	0.475		
CA74-2	1.02 (0.91–1.13)	0.762		
Tumor size	15.99 (7.56–33.82)	<0.001	2.70 (0.70–10.39)	0.147
SUVmax	6.62 (3.49–12.56)	<0.001	0.84 (0.28–2.57)	0.765
SUVmean	26.69 (9.03–78.92)	<0.001	1.86 (0.14–24.46)	0.637
TLG	2.06 (1.57–2.70)	<0.001	1.02 (0.80–1.29)	0.888
LUL	1.22 (0.66–2.26)	0.532		
LLL	1.32 (0.60–2.90)	0.495		
RUL	1.10 (0.65–1.87)	0.716		
RML	0.62 (0.24–1.60)	0.322		
RLL	0.69 (0.37–1.29)	0.244		
Nodule (solid vs GGN)	15.20 (5.43–42.53)	<0.001		
Lobulation	4.25 (2.11–8.56)	<0.001	3.03 (0.52–17.56)	0.217
Pleural change	2.40 (1.42–4.06)	0.001	0.39 (0.12–1.33)	0.133
Vacuole sign	1.27 (0.55–2.93)	0.582	0.31 (0.11–0.88)	0.028
Air bronchogram	3.49 (0.82–14.87)	0.091		
Vascular convergence	0.56 (0.20–1.59)	0.274		
Predicting Model Score	195.09 (67.46–564.17)	<0.001	11.36 (1.14–112.81)	0.038
Frozen section result	103.93 (45.93–235.18)	<0.001	48.41 (16.85–139.10)	<0.001

Data within parentheses denote the 95% CI. The Predicting Model Scores range from 0 to 1, as per the hybrid model used to distinguish between AIS/MIA and IA. Frozen section results refer to the intraoperative frozen section evaluations for patients at the clinical IA stage. Multivariate logistic regression analysis demonstrates that hybrid model prediction result is independent predictors for differentiating IA from AIS/MIA compared with CT imaging signs and intraoperative frozen section evaluation. Spiculation/calcification were excluded due to complete separation.

### Performance of the prediction models for identifying LUAD with high-risk histopathological characteristics

**Figure 3.**
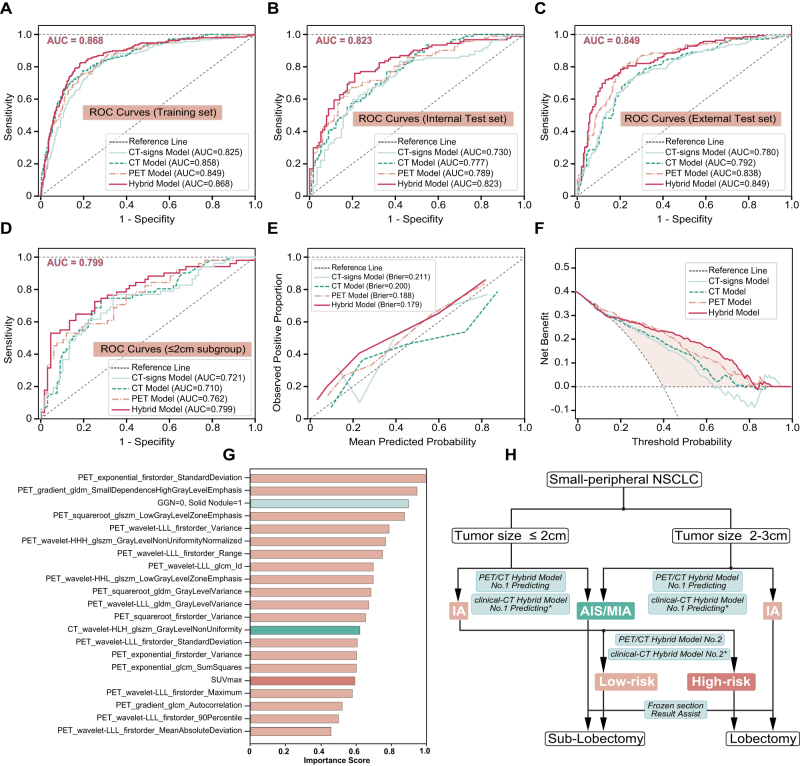
**Performance of prediction models for differentiating high-risk and low-risk histopathological characteristics in early-stage adenocarcinoma.** ROC curves of the CT-signs, CT radiomics, PET radiomics, and hybrid models for differentiating high-risk from low-risk in **(A)** training set, **(B)** internal test set, **(C)** external test set, and **(D)** tumor size ≤2 cm subgroup**. (E)** Calibration plots of the CT-signs, CT, PET, and hybrid models show the consistency between the mean predicted probability of high-risk IA and actual proportion in the internal test set. **(F)** Decision curve analysis of models for internal test set. (**G)** Importance score ranking of the hybrid model. (**H)** The two hybrid models developed in this study can guide resection strategies. Model No. 1 distinguishes AIS/MIA from IA, while Model No. 2 stratifies lesions into high-risk and low-risk histopathology.

Identified LUAD patients harboring high-risk histopathological characteristics demonstrated a preference for lobectomy rather than sub-lobectomy^[3,5,24]^. Our team engineered machine learning models utilizing the IA subtype of the training set (*N* = 638) and validated their precision using internal test set (*N* = 206) and external test set (*N* = 348) (Fig. 1). Firstly, we designed a CT-signs-based model to detect high-risk histological traits. For CT-signs model, the AUC values for the training, internal test, and external test datasets were 0.825, 0.730, and 0.780, respectively (Fig. 3A-C, Table 2).

Subsequently, CT and PET, and hybrid radiomics models were constructed. The hybrid model demonstrated superior performance with AUCs of 0.868 in the training set, 0.823 in the internal test set, and 0.849 in the external test set, outperforming individual models based on CT-signs, CT radiomics, or PET radiomics. For the subgroup of tumors ≤2 cm, the AUC of the hybrid model was 0.799 (Fig. 3D). The calibration curve for the hybrid model showed a strong concordance between predicted and observed probabilities of high-risk subtypes in the internal test set (Fig. 3E). DCA indicated that the net benefit of the hybrid model exceeded those of the other three models across nearly all threshold values in the internal test set (Fig. 3F). The importance-score ranking of hybrid model features for identifying the high-risk group was shown in Fig. 3G, highlighting that PET radiomics features play a crucial role in enhancing the accuracy of predictive models. Moreover, the weight allocation of specific indicators of CT-signs, CT, and PET models were shown in Supplemental Digital Content Figure S2E–H, available at: http://links.lww.com/JS9/F100 and Supplemental Digital Content Table S2, available at: http://links.lww.com/JS9/F100. The ultimate objective of preoperative prediction of histological subtypes of early-stage LUAD is to provide guidance for surgical resection strategies. Drawing from our developed model and the unique clinical scenario, we proposed an innovative surgical decision-making framework aimed to effectively guide clinicians and enhance patient outcomes (Fig. 3H). For patients who did not receive preoperative PET/CT, we developed and validated alternative clinical-CT hybrid models that incorporate demographics, CT-imaging-signs, and radiomics features; these alternatives retain robust discriminatory performance (Supplemental Digital Content Figure S3, available at: http://links.lww.com/JS9/F100).

Patients both in the training and test sets (derived from China) exhibited high EGFR-mutations at various stages of pathological progression (Supplemental Digital Content Figure S4A, available at: http://links.lww.com/JS9/F100). Trained hybrid model exhibits robust predictive performance for both the EGFR-mutation and EGFR-wild-type subgroups (Supplemental Digital Content Figure S4B–C, available at: http://links.lww.com/JS9/F100).

### Survival assessment

**Figure 4.**
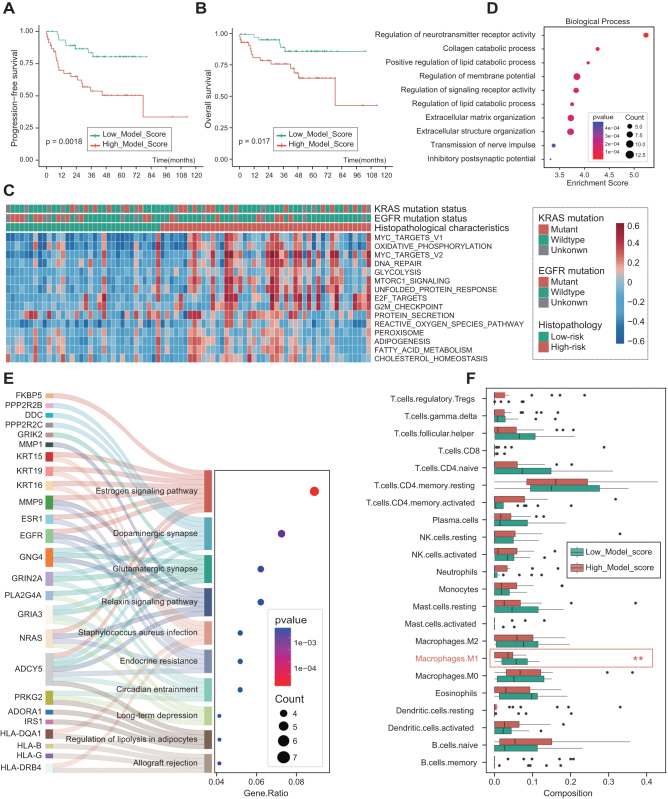
**Survival assessment and radiogenomics analysis.** Kaplan–Meier plots show the prognostic value of hybrid model predicting scores for **(A)** PFS and **(B)** OS in TCIA cohort. (**C)** Gene set variation analysis for hallmark gene sets between high-risk and low-risk histopathological characteristics. Functional enrichment analysis was conducted on differentially expressed genes. The bubble plots depict the outcomes of this analysis, utilizing **(D)** the biological process of Gene Ontology (GO) and **(E)** Kyoto Encyclopedia of Genes and Genomes (KEGG). (**F)** Distinctions in the abundance of immune cell types between high-risk and low-risk histopathological characteristics of LUAD employing ssGSEA

The medium duration of follow-up was 39.4 (0.17–112.97) months. Within the TCIA set (*N* = 94), 29 patients experienced a relapse, while 19 patients succumbed eventually. Survival rates without PFS for 1 and 2 years were 83.0% and 77.7%, respectively, whereas the OS for 1, 3, and 5 years were 90.4%, 85.1%, and 79.8%, respectively. We further scrutinized the prognostic role of the constructed hybrid model, using the median of predictive scores of hybrid model as the cut-off value, patients were categorized into two groups: High_Model_Score and Low_Model_Score groups. The Kaplan–Meier curves analysis indicated that PFS (*P* = 0.0018, Fig. 4A) and OS (*P* = 0.0170, Fig. 4B) were significantly worse in the High_Model_score than Low_Model_Score group.

### Radiogenomics analysis identifies underlying biological mechanisms correlated to high-risk histopathological characteristics of LUAD

GSVA using hallmark gene sets was conducted to compare high-risk and low-risk histopathological characteristics, with the results presented in Fig. 4C. Pathways that were upregulated in the high-risk histopathological group included MYC targets, oxidative phosphorylation, DNA repair, mTORC1 signaling, glycolysis, G2M checkpoint, and fatty acid metabolism, among others. Ultimately, a total of 191 differentially expressed genes associated with high-risk histopathology were identified. Enrichment analysis revealed that differentially expressed genes were mainly enriched in neurotransmitter receptor regulation, collagen and lipid catabolic, extracellular matrix, and estrogen signaling pathway (Fig. 4D–E). The variations in the abundance of immunocyte subsets across diverse pathological subtypes were examined through ssGSEA. The analysis revealed a significant decrease on M1-macrophage within High_Model_score group (Fig. 4F). Weighted correlation network analysis illustrated that the gene set of the salmon module exhibited a robust correlation with PET metabolic parameters (SUVmax and TLG) (Supplemental Digital Content Figure S5A-D, available at: http://links.lww.com/JS9/F100). And the top 10 biological processes of salmon modules were depicted in Supplemental Digital Content Figure S5E, available at: http://links.lww.com/JS9/F100, implying that immune cell differentiation and glucose metabolism may be correlated with PET metabolic parameters values.

## Discussion

Sublobar resection is feasible for patients afflicted with AIS and MIA. In the context of invasive LUAD, characterized by elevated tumor heterogeneity, a comprehensive evaluation of the tumor dimensions, consolidation tumor rate, location, and pathological classification is crucial to assist in the determination of an appropriate surgical approach^[2,25]^. High-risk histopathological characteristics signify unfavorable prognosis^[4,5,26]^. Thus, it is imperative to establish a noninvasive and objective methodology that can accurately predict preoperative pathological classification and high-risk histopathological characteristics to guide treatment decisions and ultimately improve patient outcomes. To our present understanding, only a limited number of research projects have devised machine learning models based on [18F]FDG PET/CT radiomics and clinical–radiological indicators to anticipate pathological classifications and histopathological high-risk LUAD.

Imaging signs of high-resolution CT and PET metabolic parameters both demonstrate robust capabilities in differentiating pathological classifications and high-risk histopathological traits in early-stage LUAD^[11,12,27]^.

In our investigation, it implies that tumor size, tumor markers, and PET metabolic parameters are effective predictors of high-grade subtype. Radiomics, with its capacity to extract imaging features objectively and quantitatively, is a promising strategy for evaluating pathological evolution. PET/CT radiomics can depict the structure and glucose metabolism of the original tumor, furnishing critical information for early detection of lung cancer, prediction of histological subtypes, response to therapy, and prognosis assessment^[28–30]^. In this research, radiomic features, predominantly those of PET images, were found to be of significant value in predicting IA and high-risk histopathological attributes.

The hybrid models demonstrated superior predictive capabilities compared to the CT models in both internal and external test sets, thereby emphasizing the potential of PET radiomic features to augment the predictive power and underscore the clinical utility of our models. This was corroborated by the Brier scores and DCA. For discriminating AIS/MIA from IA, CT models developed by *She et al* and *Huang et al* achieved AUCs of 0.89 and 0.92, respectively^[31,32]^, which were comparable with those of our CT radiomics model. Our models demonstrated strong performance in the subgroups of tumor diameter ≤2 cm and GGN. The objective is to identify smaller nodules that exhibit significant invasiveness and metastatic potential, enabling to assist surgeons in selecting surgical options and enhancing patient outcomes.

The aggregated concordance of FS and FP in this investigation paralleled those in previously published studies^[8,33,34]^. Twenty-three patients with IA subtypes were misdiagnosed as either AIS or MIA. These misclassified IA did not demonstrate high-risk subtype, indicating a favorable prognosis. Accurate diagnosis via FS serves as an effective strategy to direct resection strategies for small-peripheral lung cancers. Nevertheless, forecasting the predominant tumor pattern based solely on the FS remains a challenging endeavor^[35]^. FS provide insight into the presence of high-grade histological patterns, such as micropapillary and solid glands, with high specificity but low sensitivity, primarily due to sampling issues^[36]^. Furthermore, high-risk histopathological characteristics, such as pleural or lymphovascular invasion, cannot be identified by FS. Although the predictive power of our hybrid model for discriminating AIS/MIA from IA was inferior to that of FS, the hybrid model could still aid surgeons in making decisions before surgery with its superior specificity for identifying AIS and MIA. The findings of the multivariate logistic regression analysis revealed that the predicting score of hybrid model is an independent predictor for differentiating IA from AIS/MIA, in comparison to the intraoperative FS evaluation.

The results of the gene enrichment analysis suggested that the High_Model_score group displayed elevated activity in biological processes, including cell cycle, DNA replication, glycolysis, fatty acid metabolism, and oxidative phosphorylation. This indicated that glucose and fatty metabolism may play a critical role in the pathological evolution of lLUAD. Furthermore, this provides a potential explanation for the enhanced predictive capability of [18F]FDG PET/CT multimodal models relative to CT models.

This study presents several limitations. First, it should acknowledge that, despite the large study cohort, the results may only represent certain clinical scenarios. A distinctive pattern has recently emerged among lung cancer surgery patients in China, characterized by a rapid increase in the proportion of young, non-smoking women undergoing surgery^[37,38]^. Conversely, the TCIA dataset employed in this study was predominantly drawn from patients in the United States in previous years. It was postulated that this disparity may partially account for the significant differences in patient characteristics observed between the training and TCIA sets. Automated segmentation, rather than manual segmentation, must be developed to minimize inter-observer discrepancies. Finally, while our models demonstrate robust diagnostic accuracy, this research shares a common limitation of many artificial intelligence studies: clinical impact remains theoretically inferred rather than empirically proven. The ultimate value of these tools must be measured through their ability to improve patient outcomes or streamline clinical workflows in practice. We aspire to have the opportunity to train and validate a more generalizable model by including it in international multicenter cohort.

This study leveraged [18F]FDG PET/CT radiomics features and clinical indicators to develop dependable machine learning models that accurately distinguished AIS/MIA from IA and predicted high-risk histopathological characteristics for early-stage LUAD. Furthermore, hybrid models demonstrated exceptional performance for tumor diameter ≤2 cm and GGN subgroups. Utilizing our established hybrid models, we introduced a novel framework for surgical decision-making, designed to enhance the medical professional guidance in distinct clinical contexts for early-stage lung cancer surgery and optimize patient outcomes. Additionally, transcriptomic information was utilized to elucidate biological mechanisms and routes potentially linked to the pathological evolution of LUAD.

## Conclusion

[18F]FDG PET/CT radiomics-based models could accurately predict invasive LUAD and high-risk histopathological characteristics of early-stage LUAD, guiding optimal surgical resection.

## Data Availability

PET/CT images in this study are available from the lead contact upon request. The RNA-seq data and PET/CT image files of the NSCLC Radiogenomics collection from TCIA are available online (https://www.cancerimagingarchive.net/).

## Research registration unique identifying number (UIN)

NCT05426135.

## References

[1] Travis WD Brambilla E Noguchi M. International association for the study of lung cancer/american thoracic society/european respiratory society international multidisciplinary classification of lung adenocarcinoma. J Thorac Oncol 2011;6:244–85. 21252716 10.1097/JTO.0b013e318206a221 PMC4513953

[2] Saji H Okada M Tsuboi M. Segmentectomy versus lobectomy in small-sized peripheral non-small-cell lung cancer (JCOG0802/WJOG4607L): a multicentre, open-label, phase 3, randomised, controlled, non-inferiority trial. Lancet 2022;399:1607–17. 35461558 10.1016/S0140-6736(21)02333-3

[3] Su H Xie H Dai C. Procedure-specific prognostic impact of micropapillary subtype may guide resection strategy in small-sized lung adenocarcinomas: a multicenter study. Ther Adv Med Oncol 2020;12:1758835920937893. 32670422 10.1177/1758835920937893 PMC7336827

[4] Koike T Koike T Yoshiya K Tsuchida M Toyabe SI. Risk factor analysis of locoregional recurrence after sublobar resection in patients with clinical stage IA non-small cell lung cancer. J Thorac Cardiovasc Surg 2013;146:372–78. 23870323 10.1016/j.jtcvs.2013.02.057

[5] Eguchi T Kameda K Lu S. Lobectomy is associated with better outcomes than sublobar resection in Spread through Air Spaces (STAS)-positive T1 lung adenocarcinoma: a propensity score-matched analysis. J Thorac Oncol 2019;14:87–98. 30244070 10.1016/j.jtho.2018.09.005 PMC6309668

[6] Nitadori J-i Bograd AJ Kadota K. Impact of micropapillary histologic subtype in selecting limited resection vs lobectomy for lung adenocarcinoma of 2cm or smaller. J Natl Cancer Inst 2013;105:1212–20. 23926067 10.1093/jnci/djt166 PMC3748005

[7] Hung J-J Jeng W-J Hsu W-H Chou T-Y Huang B-S Wu Y-C. Predictors of death, local recurrence, and distant metastasis in completely resected pathological stage-I non-small-cell lung cancer. J Thorac Oncol 2012;7:1115–23. 22592210 10.1097/JTO.0b013e31824cbad8

[8] Liu S Wang R Zhang Y. Precise diagnosis of intraoperative frozen section is an effective method to guide resection strategy for peripheral small-sized lung adenocarcinoma. J Clin Oncol 2016;34:307–13. 26598742 10.1200/jco.2015.63.4907

[9] Chinese Society of Pathology. Investigation and consideration on the status of pathology departments in 3 831 hospitals of 31 provinces, municipalities and autonomous regions. Zhonghua Bing Li Xue Za Zhi 2020;49:1217–20. 33287503 10.3760/cma.j.cn112151-20200904-00695

[10] Girolami I Neri S Eccher A. Frozen section telepathology service: efficiency and benefits of an e-health policy in South Tyrol. Digit Health 2022;8:20552076221116776. 35923756 10.1177/20552076221116776 PMC9340333

[11] Shao X Niu R Jiang Z Shao X Wang Y. Role of PET/CT in management of early lung adenocarcinoma. AJR Am J Roentgenol 2020; 214:437–45. 31714848 10.2214/AJR.19.21585

[12] Bu L Tu N Wang K. Relationship between 18F-FDG PET/CT semi-quantitative parameters and international association for the study of lung cancer, American thoracic society/European respiratory society classification in lung adenocarcinomas. Korean J Radiol 2022;23:112–23. 34983098 10.3348/kjr.2021.0455 PMC8743143

[13] Niu R Shao X Shao X Wang J Jiang Z Wang Y. Lung adenocarcinoma manifesting as ground-glass opacity nodules 3 cm or smaller: evaluation with combined high-resolution CT and PET/CT modality. AJR Am J Roentgenol 2019;213:W236–W245. 31361533 10.2214/AJR.19.21382

[14] Cox VL Bhosale P Varadhachary GR. Cancer genomics and important oncologic mutations: a contemporary guide for body imagers. Radiology 2017;283:314–40. 28418819 10.1148/radiol.2017152224

[15] Hattori A Suzuki K Takamochi K. Segmentectomy versus lobectomy in small-sized peripheral non-small-cell lung cancer with radiologically pure-solid appearance in Japan (JCOG0802/WJOG4607L): a post-hoc supplemental analysis of a multicentre, open-label, phase 3 trial. Lancet Respir Med 2024;12:105–16. 38184010 10.1016/S2213-2600(23)00382-X

[16] Ye T Deng L Wang S. Lung adenocarcinomas manifesting as radiological part-solid nodules define a special clinical subtype. J Thorac Oncol 2019;14:617–27. 30659988 10.1016/j.jtho.2018.12.030

[17] Agha RA Mathew G Rashid R. Revised strengthening the reporting of cohort, cross-sectional and case-control studies in surgery (STROCSS) guideline: an update for the age of Artificial Intelligence, Prem J Sci 2025;10:100081.

[18] Bakr S Gevaert O Echegaray S. A radiogenomic dataset of non-small cell lung cancer. Sci Data 2018;5:180202. 30325352 10.1038/sdata.2018.202 PMC6190740

[19] Fedorov A Beichel R Kalpathy-Cramer J. 3D slicer as an image computing platform for the quantitative imaging network. Magn Reson Imaging 2012;30:1323–41. 22770690 10.1016/j.mri.2012.05.001 PMC3466397

[20] Blagus R Lusa L. SMOTE for high-dimensional class-imbalanced data. BMC Bioinf 2013;14:106. 10.1186/1471-2105-14-106 PMC3648438 23522326

[21] LeDell E, and Poirier S. H2O AutoML: Scalable Automatic Machine Learning. 7th ICML Workshop on Automated Machine Learning (AutoML), July (2020). Accessed 26 September 2025. https://www.automl.org/wp-content/uploads/2020/07/AutoML_2020_paper_61.pdf.

[22] Charoentong P Finotello F Angelova M. An-cancer immunogenomic analyses reveal genotype-immunophenotype relationships and predictors of response to checkpoint blockade. Cell Rep 2017;18:248–62. 28052254 10.1016/j.celrep.2016.12.019

[23] Tang D Chen M Huang X. SRplot: a free online platform for data visualization and graphing. PLoS One 2023;18:e0294236. 37943830 10.1371/journal.pone.0294236 PMC10635526

[24] Akamine T Wakasu S Matsubara T. Is sublobar resection feasible for high-risk pathologic stage I non-small cell lung cancer? Ann Surg Oncol 2025;32:4161–72. 39681715 10.1245/s10434-024-16700-z

[25] Altorki N Wang X Kozono D. Lobar or sublobar resection for peripheral stage IA non-small-cell lung cancer. N Engl J Med 2023;388:489–98. 36780674 10.1056/NEJMoa2212083 PMC10036605

[26] Emoto K Eguchi T Tan KS. Expansion of the concept of micropapillary adenocarcinoma to include a newly recognized filigree pattern as well as the classical pattern based on 1468 stage I lung adenocarcinomas. J Thorac Oncol 2019;14:1948–61. 31352072 10.1016/j.jtho.2019.07.008 PMC8785415

[27] Eriguchi D Shimada Y Imai K. Predictive accuracy of lepidic growth subtypes in early-stage adenocarcinoma of the lung by quantitative CT histogram and FDG-PET. Lung Cancer 2018;125:14–21. 30429012 10.1016/j.lungcan.2018.08.027

[28] Monaco L De Bernardi E Bono F. The “digital biopsy” in non-small cell lung cancer (NSCLC): a pilot study to predict the PD-L1 status from radiomics features of [18F]FDG PET/CT. Eur J Nucl Med Mol Imaging 2022;49:3401–11. 35403860 10.1007/s00259-022-05783-z

[29] Dissaux G Visvikis D Da-Ano R. Pretreatment 18F-FDG PET/CT radiomics predict local recurrence in patients treated with stereotactic body radiotherapy for early-stage non-small cell lung cancer: a multicentric study. J Nucl Med 2020;61:814–20. 31732678 10.2967/jnumed.119.228106

[30] Mu W Jiang L Zhang J. Non-invasive decision support for NSCLC treatment using PET/CT radiomics. Nat Commun 2020;11:5228. 33067442 10.1038/s41467-020-19116-x PMC7567795

[31] Huang L Lin W Xie D. Development and validation of a preoperative CT-based radiomic nomogram to predict pathology invasiveness in patients with a solitary pulmonary nodule: a machine learning approach, multicenter, diagnostic study. Eur Radiol 2022;32:1983–96. 34654966 10.1007/s00330-021-08268-z PMC8831242

[32] She Y Zhang L Zhu H. The predictive value of CT-based radiomics in differentiating indolent from invasive lung adenocarcinoma in patients with pulmonary nodules. Eur Radiol 2018;28:5121–28. 29869172 10.1007/s00330-018-5509-9

[33] Zhu E Xie H Dai C. Intraoperatively measured tumor size and frozen section results should be considered jointly to predict the final pathology for lung adenocarcinoma. Mod Pathol 2018;31:1391–99. 29752477 10.1038/s41379-018-0056-0

[34] Zhang Y Deng C Fu F. Excellent prognosis of patients with invasive lung adenocarcinomas during surgery misdiagnosed as atypical adenomatous hyperplasia, adenocarcinoma in situ, or minimally invasive adenocarcinoma by frozen section. Chest 2021;159:1265–72. 33197404 10.1016/j.chest.2020.10.076

[35] Trejo Bittar HE Incharoen P Althouse AD Dacic S. Accuracy of the IASLC/ATS/ERS histological subtyping of stage I lung adenocarcinoma on intraoperative frozen sections. Mod Pathol 2015;28:1058–63. 26022456 10.1038/modpathol.2015.71

[36] Yeh Y-C Nitadori JI Kadota K. Using frozen section to identify histological patterns in stage I lung adenocarcinoma of ≤ 3 cm: accuracy and interobserver agreement. Histopathology 2015;66:922–38. 24889415 10.1111/his.12468 PMC4536823

[37] Luo G Zhang Y Rumgay H. Estimated worldwide variation and trends in incidence of lung cancer by histological subtype in 2022 and over time: a population-based study. Lancet Respir Med 2025;13:348–63. 39914442 10.1016/S2213-2600(24)00428-4

[38] Han B Zheng R Zeng H. Cancer incidence and mortality in China, 2022. J Natl Cancer Cent 2024;4:47–53. 39036382 10.1016/j.jncc.2024.01.006 PMC11256708

